# The effect of ejection fraction on mortality in Coronary Artery Bypass Grafting (CABG) patients

**DOI:** 10.12669/pjms.36.7.3266

**Published:** 2020

**Authors:** Nabil Iftikhar Awan, Azam Jan, Mujeeb Ur Rehman, Narmeen Ayaz

**Affiliations:** 1 Dr. Nabil Iftikhar Awan MBBS. Post-Graduate Resident, Department of Cardiovascular & Thoracic surgery, Rehman Medical Institute, Peshawar, Pakistan; 2 Dr. Azam Jan, ABTS. Head of Department, Department of Cardiovascular & Thoracic surgery, Rehman Medical Institute, Peshawar, Pakistan; 3 Dr. Mujeeb Ur Rehman, MS. Senior Medical Officer, Department of Cardiovascular & Thoracic surgery, Rehman Medical Institute, Peshawar, Pakistan; 4 Dr. Narmeen Ayaz, MBBS. House-Officer, Department of Cardiovascular & Thoracic surgery, Rehman Medical Institute, Peshawar, Pakistan

**Keywords:** Coronary Artery Bypass Graft, Ejection Fraction, Mortality, Cardiac surgery, Intra-aortic Balloon Pump

## Abstract

**Background and Objectives:**

Patients with low ejection fraction undergoing isolated CABG surgery are at a higher risk for postoperative complications and mortality. This study was conducted to evaluate the impact of ejection fraction on the outcome of isolated Coronary Artery Bypass Grafting (CABG).

**Methods:**

Between July, 2017 to May, 2019 total 1214 patients underwent isolated CABG. Patients were divided into three groups based on their pre-operative Ejection Fraction (EF). Group-I included 625 patients with EF >50% [Normal EF], Group-II included 484 patients with EF 35-50% [Mild to Moderately Reduced EF], and Group 3 included 105 patients with EF<35% [Severely Reduced EF].

**Results:**

The mean age of Group-I was 57.58 ± 9.206, Group-II was 58.38±9.124 and Group-III was 58.81± 8.663. The male gender was the predominant gender in all three groups: 194(41.1%) in Group-I, 201(52.6%) in Gp2, 52 (61.9%) in Group-III. 231(36.9) patients in Group-I, 234(48.3)in Group-II and 59(56.2) in Group-III had raised creatinine pre operatively. 5(0.8%) patients in Group-I, 2(0.4%) in Group-II and 3(2.9%) in Group-III had history of CVA. Hypertension was present in approximately 60% of all our patients. In the per-operative period 20(3.2%) patients in Group-I required an IABP as compared to 73(15.1%) in Group-II and 41(39.0%) in Group-III. The mean post-operative mortality in Group-I was 19 (3%), Group-II was 24(5.0%) and low EF group was 9(8.6%).

**Conclusions:**

The results clearly indicate that worsening pre-operative ejection fraction is associated with a higher mortality post-operatively in patients undergoing isolated CABG. In addition, use of IABP increases as pre-operative LVEF decreases.

**Definitions:**
*PERFUSION TIME: total time on CPB machine.
*CROSS CLAMP TIME: Total time that ascending aorta was CROSS clamped.
*STROKE: Defined as presence of neurological deficit, findings on CT scan and confirmed by a Neurology consultant.
*RE-OP: Re-operation during index admission.

## INTRODUCTION

According to the Global Burden of Disease Study data from 2017, Ischemic Heart Disease is responsible for 8.93 million deaths annually which accounts for approximately 15.96% of all deaths worldwide.^1^ However large this number sounds, it was the advent of goal directed medical therapy, PCI and CABG that brought it down from an even larger number. In spite of this, it has recently been found that the incidence of Coronary Artery Disease has been on the rise in our province and therefore special attention needs to be given on not only its prevention but also its management.^2^

Since its inception more than 40 years ago, CABG surgery has been an important tool for reducing symptoms and/or improving survival in patients suffering from coronary artery disease. However, CABG surgery is not entirely risk-free. CABG surgery currently boasts an all-cause mortality rate of 1% in patients with normal LVEF which goes up to 7% in patients with severely reduced EF.^3^ A combination of patient selection, improved surgical technique and pre-operative optimization has played a significant role in improving mortality after CABG surgery.^4^ Nowadays, cardiac support devices in the form of IABP, LVAD, Impella and TAH are being used as targeted therapy for heart failure and thus have further enhanced survival.

A lot of research has been done to identify the risk factors that contribute to mortality after CABG surgery. We now know that a reduced LVEF is a risk factor for post-CABG mortality.^4^ We wanted to find a direct relationship between LVEF and CABG outcomes, specifically in-hospital mortality.

## METHODS

This study was conducted after approval by The Ethical Review Committee of our hospital with Ref No: RMI/RMI-REC/Study Approval/33, June 22, 2020 and consent of the patients. The center where this study was conducted, a cardiac surgery database is maintained. This practice allows us to prospectively collect the pre-operative, per-operative and post-operative data of all patients undergoing cardiac surgery.

From this database, we extracted the data of all patients that had undergone isolated CABG at our setup between July, 2017 and May, 2019. We excluded all those patients who had a concomitant surgical procedure performed like valve replacement, repair or ASD/VSD closure. Total 1214 patients fulfilled our inclusion criteria.

Based on their LVEF, we divided these patients into 3 groups according to the American echocardiographic association. Group-I had 625 patients with normal LVEF. Group-II had 484 patients with mild to moderately reduced LVEF, group 3 had 105 patients with severely reduced LVEF.

For these groups, we divided the collected data into three further groups: the pre-operative, per-operative, and post-operative group. The data comprised of age, gender, NYHA class, CCS class, COPD, Dyslipidemia, diabetes mellitus, myocardial infarction, LMS disease and stroke. The in-hospital mortality is defined as mortality within index hospital admission during the hospital stay post operatively.

All the necessary preoperative radiological and laboratory investigations were performed including chest x-ray, ECHO, ECG, angiography. Carotid Doppler ultrasound was performed in selected patient (e.g. age >50, LMS disease, smokers, BMI >30). The patients underwent routine on-pump CABG with regular anesthetic technique. We used the Left Internal Mammary artery as the only arterial conduit and the Retrograde Saphenous Vein Graft for our venous conduit. For data entry and analysis we used the SPSS software. For statistical calculation we used the Chi-squared test. P-value less than 0.05 was considered to be significant.

## RESULTS

A total of 1214 patients were included in the study: 625 patients belonged to Group-I, 484 patients belonged to Group-II, and 105 patients belonged to group 3. The average age in years of all the patients combined was 58.25 yrs. The average age of patients in Group-I was 57.58, 58.38 in Group-II, and 58.8 in group 3. A total of 77.2% of all the patients were males. Group-I had 75.5% males while Group-II had 78.9% and group 3 had 80% males. Table-I shows baseline characteristics of the patients undergoing isolated CABG. In comparison of all three groups, male gender was dominant and most patients had normal Ejection Fraction pre-operatively. Patients with severely reduced EF had a higher incidence of dyslipidemia, cerebrovascular disease, hypertension, diabetes, renal insufficiency with high creatinine (normal creatinine in females: 0.5-0.9, normal creatinine in males: 0.7-1.2). As compared to the patients with normal EF, history of myocardial infarction and use of IABP intra operatively was higher in severely reduced EF group. The result of IABP use was significant between the groups (the p value < 0.00001).

**Table-I:** Baseline characteristics of patients undergoing CABG.

Variables	Normal EF (>50) (N=625)	Mild to Moderate Ef (35-50%) (N=484)	Severly Reduced EF (<35%) (N=105)
Age (Y) (Mean ± SD)	57.58 ±9.206	58.38±9.124	58.81±8.663
Male SEX (%)	472(75.5)	382(78.9)	84(80.0)
Female SEX (%)	153(24.5)	102(21.1)	21(20.0)
*Deranged Renal Profile*			
Pre-op Raised Creatinine	231(36.9)	234(48.3)	59(56.2)
Pre-OP Raised Creatinine in Males (%)	194(41.1)	201(52.6)	52(61.9)
*Dyslipidemia*			
With Statin Use (%)	306(49.0)	233(48.1)	39(37.1)
Without Statin Use (%)	3 (0.5)	2(0.4)	1(1.0)
*Pulmonary Disease*			
Chronic Lung Disease (%)	2(0.3)	2(0.4)	0
*Cerebrovascular Disease*			
Previous CVA (%)	5(0.8)	2(0.4)	3(2.9)
*Carotid disease*			
Non invasive>75 %(%)	4(0.6)	3(0.6)	0
Hypertension (%)	398(63.7)	302(62.4)	69(65.7)
Diabetes (%)	227(36.3)	220(45.5)	53(50.5)
*Prior cardiac procedure*			
First (%)	595(95.2)	461(95.2)	102(97.1)
Second (%)	10(1.6)	8(1.7)	1(1.0)
Third (%)	0	1(0.2)	0
Previous pci (%)	14(2.2)	17(3.5)	4(3.8)
Prior mi (%)	74(11.8)	83(17.1)	23(21.9)
*Pre-operative medications*			
Beta blockers (%)	324(51.8)	267(55.2)	48(45.7)
Ace inhibitors (%)	73(11.7)	103(21.3)	20(19.0)
Nitrates (%)	245(39.2)	195(40.3)	35(33.3)
Warfarin (%)	0	5(1.0)	5(4.8)
Aspirin (%)	294(47.0)	223(46.1)	42(40.6)
Statin (%)	237(37.9)	309(63.8)	54(51.4)
Antiplatelet <5 days pre-op (%)	324(51.8)	183(37.8)	36(34.3)
Oral hypoglycemic (%)	73(11.7)	124(25.6)	36(34.3)
*No of diseased coronary vessels*			
Single vessel (%)	0	1(0.2)	0
Double vessel (%)	41(6.6)	40(8.3)	6(5.7)
Triple vessel (%)	581(93.0)	441(91.1)	98(93.3)
Left main disease >50% (%)	53(8.5)	45(9.3)	13(12.4)
*Co-existing valvular conditions*			
Aortic stenosis (%)	2(0.3)	3(0.6)	0
Mitral stenosis (%)	1(0.2)	3(0.6)	0
Aortic insufficiency (%)	61 (9.76)	57(11.77)	21(20)
Mitral insufficiency (%)	161(25.76)	262(54.13)	86(81.90)
Tricuspid insufficiency (%)	49(7.84)	98(20.24)	42(40)
Pulmonic insufficiency (%)	9( 1.44)	1(0.20)	1(0.95)

*Normal Creatinine in Females: 0.5-0.9 *Normal Creatinine in Males: 0.7-1.2

Most of the patients included in this study were using cardiac medications as shown in Table-I about 12.4 % of the patients of severely reduced EF group have left main artery disease (> 50% blocked) and majority of the patients had triple vessel disease diagnosed on pre-operative Cardiac Catheterization (Table-II). The patients who had additional valvular disease were excluded from the study. Carotid disease was only evaluated for high risk patients including the patients who had left main stem disease and age > 60. Intra-Aortic Balloon Pump was used in 39% of patient with severely reduced EF which was higher as compared to the normal and moderately reduced EF groups (Table-III). Mean number of blood transfusions in low EF group was 0.64 with SD of 0.483. 96.2% of the patients in severely reduced EF group underwent intra-operative and post-operative transfusions of blood products that included fresh frozen plasma, platelets and packed cells RBCs.

**Table-II:** Pre-operative clinical status.

Variables	Normal Ef (>50) (n=625)	Mild to moderate Ef (35-50%) (n=484)	Severly reduced Ef (<35%) (n=105)
*Nyha classification*			
Nyha class 1(%)	6(1.1)	9(1.9)	1(1.0)
Nyha class 2(%)	71(11.4)	51(10.5)	3 (2.9)
Nyha class 3 (%)	252(40.3)	198(40.9)	50(47.6)
Nyha class 4 (%)	221 (35.4)	170(35.1)	38 (36.2)
*Ccs classification*			
Ccs class 1 (%)	54(8.6)	46(9.5)	7(6.7)
Ccs class 2 (%)	246(39.4)	177(36.3)	44(41.9)
Ccs class 3 (%)	272(43.5)	208( 43.3)	44(41.9)
Ccs class 4 (%)	27(4.3)	33(6.8)	6(5.7)

**Table-III:** Comparison of Operative Variables.

Variables	Normal Ef (>50) (N=625)	Mild to moderate Ef (35-50%) (n=484)	Severly Reduced Ef (<35%) (N=105)	p-Value
*Operative*				
Perfusion Time (Mean ± SD)	94.78 ±25.036	97.83±25.781	95.84±20.786	-
Cross clamp time (Mean ± SD)	52.93 ±15.241	54.08±16.831	51.26±15.887	-
IABP (%)	20(3.2)	73(15.1)	41(39.0)	0.00001
*Coronary Bypass*				
Lima (%)	559(89.4)	402(83.1)	65(61.9)	-
Avg no. of vein grafts (Mean ± SD)	2.49±0.661	2.55±0.699	2.70±0.678	-
*Blood products*				
Avg. Number of blood transfusion/patient (mean ± SD)	2.49 ±0.661	0.53±0.500	0.64±0.483	-
Total Number of Patient Requiring Intra/Post OP Blood Products (%)	556(89.0)	438(90.5)	101(96.2)	-
*Complications*				
Re-op (%)	35(5.6)	22(4.5)	2(1.9)	0.243
Post-op stroke (%)	4(0.6)	4(0.8)	1(1.0)	0.905
Prolonged ventilation >24 hours (%)	10(1.6)	8(1.7)	3(2.9)	0.649
AF	7(1.1)	7(1.4)	0	-
Prolonged ICU Stay > 48 Hours (%)	34(0.59)	32(0.64)	10(0.9)	0.256
In Hospital Mortality (%)	19(3.0)	24(5.0)	9(8.6)	0.022

2.9% of the patients in low EF group had prolonged ventilation, 5.6% of the normal EF group were re-opened within 48 hours post operatively for bleeding/tamponade. Complication of post-operative stroke during the hospital stay was highest in reduced EF group that is 1%, prolong ICU stay that is more than 48 hours was also common in same group (0.9%) discharge to home ratio was significantly lower in patients with reduced EF (91.4%). In hospital post-operative mortality was highest in patients with reduced EF group that is 8.6%, 5% in moderate EF group and 3%in normal EF group making the result significant: p-value <0.022324.

## DISCUSSION

Coronary artery bypass grafting has been shown to offer greater benefit in patients with poor left ventricular function. El Alderman et al. showed CABG improved survival significantly in patients who had low ejection fractions when compared with medical treatment alone.^5^,^6^ Specifically, patients with ejection fractions below 26% had a greater survival benefit after surgery, translated as a 63% 5-year mortality rate when compared with the 43% 5-year mortality of medical therapy.^4^,^5^ In addition, the seven year survival of patients with EF<50% who had undergone CABG (84%) was significantly higher than those patients who were on guideline directed medical therapy (70%).^7^ This finding was also confirmed on the 10-year results of the CASS study.^6^ Multiple studies have also shown that CABG surgery resulted in considerable improvements in both the NYHA angina class and the left ventricular ejection fraction.^9^ It is therefore established that CABG in this population of severely reduced ejection fraction is a viable option with better results than other modes of treatment.^8^

However, it is also a known fact that CABG surgery in this group of patients who have low ejection fraction comes at a higher risk of post-operative mortality. A review that compared the in-hospital mortality rates of patients undergoing CABG surgery noted that patients who had an EF <35% had a mortality rate that was six times higher than that of patients with EF>50%.^4^ Mortality rates of patients with low ejection fraction have been reported as high as 11% by Carr et al.^9^ Others, more recently have registered mortality rates to be around 4% as mentioned in the review of the New York State Database for CABG surgery.^3^ This single digit mortality in the low EF group is a product of, among other factors, better surgical technique, improved cardiac anesthesia, post-operative care and most importantly a refined understanding of the management of LCOS and the ever significant inclusion of Extracorporeal Membrane Oxygenators (ECMO) and Left Ventricular Assist Devices (LVAD).

In this review of our own database, in-hospital mortality rates of patients with reduced ejection fraction was considerably higher than those with a normal or moderate ejection fraction. We registered rates of 8.6% in the low EF group as compared to 5% and 3% in the moderately reduced and normal ejection fraction groups respectively. We understand that these numbers are higher than those reported by other multi-center trials. In our center, where this study was conducted, we do not have the availability of LV support devices in the form of ECMO or LVAD. In addition, there aren’t any centers in our country who are doing heart transplants. A proportion of our mortalities can be theoretically prevented if we can institute any of the above mentioned therapies. However, comparing our hospital results with other hospitals in our region revealed no significant difference as a recent study conducted by one of the largest cardiac centers in our country revealed a mortality rate of 3.8% which was similar to our mortality rate of 3%.^10^

The use of intra-aortic balloon pump also displayed a similar trend. It was demonstrated in a recent study that patients who had low ejection fractions (EF<35%) had a higher rate of IABP application (25.8%) when compared to those with higher ejection fractions (EF 36-50%) (9.2%).^11^

In our study population, we replicated similar results. The IABP rate in the low EF group was considerably greater at 36% when compared to the normal EF group in whom IABP rate was merely 3.6%. A very famous institute in our country has reported lower rate of IABP use (17.9%) in patients with severely reduced Ejection Fractions. This may be due to variation in the thresholds for IABP amongst various centers.^12^

In our data, we found no statistically significant difference between groups in the rate of stroke, post-operative prolonged ventilation, or prolonged ICU stay. Stamou in their analysis of the predictors of post-operative stroke in CABG patients have also not stated pre-operative low ejection fraction as an independent risk factor.^13^

### Limitation of study

This study has attempted to evaluate the effect of ejection fraction on mortality in patients undergoing CABG in our population. However, there are a number of limitations to our study.

When identifying the post-operative outcomes we have evaluated the in-hospital mortality only. Further research can be targeted to extract the effect of ejection fraction on long term mortality. Furthermore, at our set-up we do not have Extra-corporeal Membrane Oxygenators, Left Ventricular Assist Devices or the facility of heart transplant. All of these have the ability to reduce the mortality and therefore the mortality rates stated in this study cannot be duplicated in a center that will have the above facilities.

In addition, we understand that in a number of patients the reduced ejection fractions pre-operatively was probably due to hibernating myocardium that theoretically would have improved significantly immediately after revascularization. Further studies on this subject need to be done on this subject to account for this phenomenon. Moreover, the cause of death was not identified in our study. More work needs to be done on this data to find out the attributable cause of death so we can more accurately state the effect ejection fractions have on mortality in CABG patients.

## CONCLUSION

Worsening pre-operative ejection fraction is associated with a higher mortality post-operatively in patient undergoing isolated CABG. In addition, the use of IABP is also dependent on the pre-operative ejection fraction.

### Authors Contribution:

**NIA:** Data collection, manuscript writing and editing, statistical analysis.

**NA and MUR:** Data collection and manuscript writing.

**AJ:** Conceived, designed, review and final approval of manuscript..
